# Inflammation Pharmacological Reaction and YMDD Mutational Patterns in Lamivudine Therapeutics Hepatitis B Virus

**DOI:** 10.3389/fphar.2021.648170

**Published:** 2021-04-15

**Authors:** Hongcan Liu, Zemin Wan, Lanhui She, Yajuan Zhu, Zhiliang Cai, Bin Wu, Qizhen Zhuang, Peifeng Ke, Xinzhong Wu, Zhuo Li, Xianzhang Huang

**Affiliations:** ^1^ Department of Laboratory Medicine, The Second Affiliated Hospital of Guangzhou University of Chinese Medicine, Guangzhou, China; ^2^ Department of Infectious Diseases, Guangzhou Women and Children’s Medical Center, Guangzhou, China; ^3^ Department of Ultrasound, The First Affiliated Hospital of USTC, Division of Life Sciences and Medicine, University of Science and Technology of China, Hefei, China; ^4^ Genetic Testing Lab, The Second Affiliated Hospital of Guangzhou University of Chinese Medicine, Guangzhou, China

**Keywords:** lamivudine therapeutics, inflammation pharmacological reaction, chronic hepatitis B, YMDD mutation, molecular mechanisms, biochemical response

## Abstract

**Background/Aims**: Emergence of tyrosine-methionine-aspartate-aspartate (YMDD) motif in reverse transcriptase is a serious problem in chronic hepatitis B(CHB) patients after Lamivudine (LAM) therapy. However, the relationship between inflammation pharmacological reaction and YMDD mutational patterns of CHB has not been well-characterized. The aim of this study was to investigate the inflammation pharmacological reaction and different YMDD mutants patterns of CHB patients.

**Methods**: We investigated the inflammation pharmacological reaction and YMDD mutational patterns through biochemical, serological and virological detection among 83 CHB patients, including 25 YMDD mutants, 25 under detection, and 33 control patients without YMDD mutants.

**Results**: Prevalence of YMDD mutation patterns is different. Among 25 YMDD mutants patients, YIDD was the dominant mutation (72%), followed YVDD (16%) and the hybrid YIDD + YVDD (12%). The time course during the YMDD mutations was also different. 52.4% patients developed the mutation less than 12 months after the LAM therapy. Serum hepatitis B virus (HBV) DNA level in patients with YMDD mutants were significantly higher than that in control and negative groups. Serum HbsAg and HbeAg in patients with YMDD mutants were also higher than those in control and negative groups, despite no significant difference was found forserum HbeAb. ALT and AST levels were also significantly higher in mutants group.

**Conclusions**: Illuminating inflammation pharmacological reaction and YMDD mutational patterns of CHB during pathological process may have implications for future therapy in YMDD mutation patients. This may have impact on the choice of treatment strategies for lamivudine-resistant HBV.

## Introduction

Hepatitis B virus (HBV) infection has long been a serious public health problem all over the world (European Association for the Study of the Liver, 2017). About two billion people are infected with HBV, and more than 350 million have become chronic carriers (Schweitzer et al., 2015). HBV infection have a high risk of causing serious liver diseases, including liver fibrosis, cirrhosis and hepatocellular carcinoma (HCC) (Samal et al., 2012). HCC is the fifth most common cancer and major cause of cancer death (de Lope et al., 2012). Antiretroviral treatment is the main clinical treatment of HBV (European Association for the Study of the Liver, 2017). Lamivudine (LAM), the first nucleoside analogue approved for treatment of CHB (Lai et al., 1998), has been widely used in clinic because of its high effectiveness (Chen et al., 2014). However, LAM treatment could cause LAM resistance in some HBV cases (Lai et al., 1998). The cause of the resistance was the mutation of the amino acid substitutions in the highly conserved tyr-met-asp-asp (YMDD) motif (Ling et al., 1996). The most common mutational pattern is methionine substitution at amino acid position-204 to either isoleucine (rtM204I, YIDD mutant) or valine (rtM204V, YVDD mutant) (Hashimoto et al., 2010). YMDD mutation has serious interference and can lead to failure during LAM treatment in CHB (Zoulim and Locarnini, 2012).

YMDD mutation is also an independent risk factor for HCC in liver cirrhosis patients (Yang et al., 2013). Many studies have reported the incidence and characteristic of spontaneous YMDD-motif mutation in LAM-naive CHB patients. Kim, et al. found the baseline YMDD mutation patterns were as follows: rtM204I, 45 (57.7%); rtM204V, 26 (33.3%); and rtM204I/V, 7 (9.0%) (Kim et al., 2012). Among the 51 patients, Chen, et al. found 30 (59%) had one single YMDD variant: including 27 (53%) rtM204I, 1 (2%) rtM204V, and 2 (4%) rtL180M Chen et al. (2004). The YMDD mutational incidence was different in a study-dependent manner (Lai et al., 2003; Yuen et al., 2003; Kim et al., 2012). The emergence of YMDD mutants considerably reduces the viral susceptibility to LAM treatment. The loss of efficacy generally increases over time post LAM treatment, from 14–47% after 1 year to 26–71% after 2 years, 49–57% after 3 years, and 67% after 4 years (Tsubota, 2006). Liu, et al. found that the majority of the cases (42/63, 66.6%) with YMDD mutants were detected between 12 and 24 months post therapy (Liu et al., 2005). To the best of our knowledge, there is limited data in terms of the proportion and persistent time during YMDD mutants pathological process. Many studies studied the relationship between YMDD mutations patterns and HBV-DNA levels, HBeAg status, and serum alanine aminotransferase (ALT) levels in patients after receiving LAM therapy for CHB. Patients with YMDD variants lack clinical response to LAM therapy, showing a significant increase of the HBV DNA and ALT levels (Lai et al., 2003). It has also been found that YMDD mutants have less replication competence and were associated with less aggressive liver disease (Zöllner et al., 2000). Wu et al. reported that HBV genotype, HBV-DNA levels, and HBeAg status at baseline were the independent factors associated with the emergence of YMDD mutations among patients receiving LAM therapy for CHB (Wu et al., 2012). Lee et al. has demonstrated YMDD mutants occur throughout the course of LAM therapy irrespective of occurrence of viral DNA breakthrough (Lee et al., 2006). Patients withdiscontinued LAM therapy have increased frequency of flare-ups and higher ALT peaks (Chen et al., 2004). However, some studies were inconsistent, or even contradictory. Therefore, the influence of LAM-resistant mutations on the antiviral effect of CHB treatment remains to be elaborated. The aim of this study was to analyze the patterns and incidence of LAM-resistant mutations, and to analyze the influence on biochemical and virological responses after YMDD mutations in CHB patients with LAM-resistant.

## Materials and Methods

### Patients

A total of 83 patients with CHB (58 males and 25 females) were recruited from the inpatient departments of Guangdong provincial hospital of Chinese Medicine from 2012 January to 2018 December. CHB was diagnosed according to the diagnostic standard from the Chinese National Program for Prevention and Treatment of Viral Hepatitis. These patients received lamivudine (LAM) therapy (orally, 100 mg/day) according to the Physician and their clinical characteristics were showed in Table 1. All the patients were divided into three groups, including control group: 33 patients’ YMDD gene were wild type; Mutation group: 25 patients who had developed YMDD mutants after LAM therapy; Negative group: 25 patients without detected mutants. Serum samples were collected from patients, and the biochemical, serological and virological data were performed with serum samples from each patient on the same day. Patients who had received interferon-α or other nucleotide analogs, or those co-infected with hepatitis C, hepatitis D and human immunodeficiency virus (HIV) were excluded from the study. Written consent was obtained from all patients. The procedures were approved by the local ethics committee and are in accordance with the Helsinki Declaration.

**TABLE 1:** Clinical characteristics.

Characteristics	Median ±SD			Total
	Control group (N 33)	Mutation group (N 25)	Negative group (N 25)
Male (N,%)	22 (66.67%)	18 (72%)	18 (72%)	58 (69.88%)
Female (N,%)	11 (33.33%)	7 (28%)	7 (28%)	25 (30.12%)
Age	49.03 ± 11.45	54.92 ± 14.95(*p* < 0.05)	54.60 ± 12.71(*p* = 0.09)	
YIDD mutation (N%)		18 (72%)		
YVDD mutation (N%)		4 (16%)		
Hybrid mutation (N%)		3 (12%)		
HBV DNA (log_10_IU/mL)	3.10 ± 0.52	5.77 ± 1.42(*p* < 0.01)	2.63 ± 0.81(*p* < 0.01)	
HBsAg(IU/mL)	4,898.89 ± 2,105.98	6,468.75 ± 2,306.99(*p* < 0.05)	6,024.85 ± 2,465.38(*p* = 0.12)	
HBeAg(IU/mL)	0.10 ± 0.03	1.23 ± 2.26(*p* < 0.05)	0.95 ± 1.77(*p* < 0.05)	
HBeAb (IU/mL)	0.009 ± 0.02	0.27 ± 0.39(*p* = 0.09)	0.45 ± 0.53(*p* < 0.05)	
HBcAb (IU/mL)	0.007 ± 0.002	0.006 ± 0.002(*p* < 0.05	0.006 ± 0.002(*p* < 0.01	
AST (U/L)	36.93 ± 15.60	55.45 ± 46.46(*p* < 0.01)	41.46 ± 18.76(*p* = 0.4)	
ALT (U/L)	33.29 ± 15.88	54.82 ± 58.15(*p* < 0.05)	36.50 ± 20.24(*p* = 0.5)	

Control group: patients’ YMDD gene was wild type. Mutation group: patients were developed YMDD mutants after LAM therapy. Negative group: patients’ YMDD mutants did not detect. HBV DNA: hepatitis B Viral DNA, HBsAg: hepatitis B surface antigen, HBeAg: hepatitis B e antigen, HBeAb: hepatitis B e antibody, HBcAb: hepatitis B core antibody, AST: aspartate aminotransferase, ALT: alanine aminotransferase.

### Detection of YMDD Mutant Types

YMDD mutant types were determined by the ABI 7500 Real-Time qPCR System (United States), according to the manufacturer’s instructions and melting curve assay with the use of the care HBV mutation PCR assay and distinguished by melting temperature value.

### Quantitation of Serum HBV DNA

Hepatitis B viral DNA from serum samples were extracted by using Desoxyribo Nucleic Acid Isolation Kit provided by Da An Gene Co., Ltd. Of Sun Yat-sen University as per the manufacturer’s instructions. HBV DNA were performed using the ABI 7500 Real-Time qPCR System (United States), according to the manufacturer’s instructions (dynamic range 1*102–1*1010 IU/ml).

### Hepatitis B Virus Marker

Quantitative serum HBV markers including HBsAg, HBsAb, HBeAg, HBeAb and HBcAb assay were quantified using routine automated immune analyzers (Cobas e602, obtaining ISO15189 certification), according to the manufacturer’s instructions respectively. Quantitative HBsAg levels were reported in IU/ml, with a dynamic range of 0.05–1000 IU/ml. Samples were dilutions of one in 100 or one in 1,000 when the maker levels were above the range. The diluent is non-disturb for the objective biochemical, serological and virological detection.

### Biochemical Parameters

Liver biochemical test including alanine aminotransferase (ALT), as-partate aminotransferase (AST) were performed using routine automated analyzers (Cobas 8,000, obtaining ISO15189 certification), according to the manufacturer’s instructions respectively.

### Statistical Analysis

All data are presented as mean ± SD. Demographic data were analyzed using descriptive statistical tests and performed by SPSS software package, version 20.0 and GraphPad Prism 5. The different Comparison between two groups were performed by the unpaired *t*-test and the numeration data were analyzed by the χ (Schweitzer et al., 2015) test. A difference with *p* < 0.05 was considered statistically significant.

## Results

### Patient Clinical Characteristics

Eighty three CHB patients for LAM therapy were recruited. All patients were from the inpatient departments of Guangdong provincial hospital of Chinese Medicine. The classification of patients into respective groups were: control group (N = 33), mutation group (N = 25), negative group (N = 25). The baseline of patients clinical characteristics were presented in Table 1. There were more males (69.88%) than females (30.12%) in the study group (56%). The control group patients were younger than mutation group and negative group (*p* < 0.05).

### Prevalence of YMDD Mutation Patterns After LAM Therapy

**FIGURE 1:**
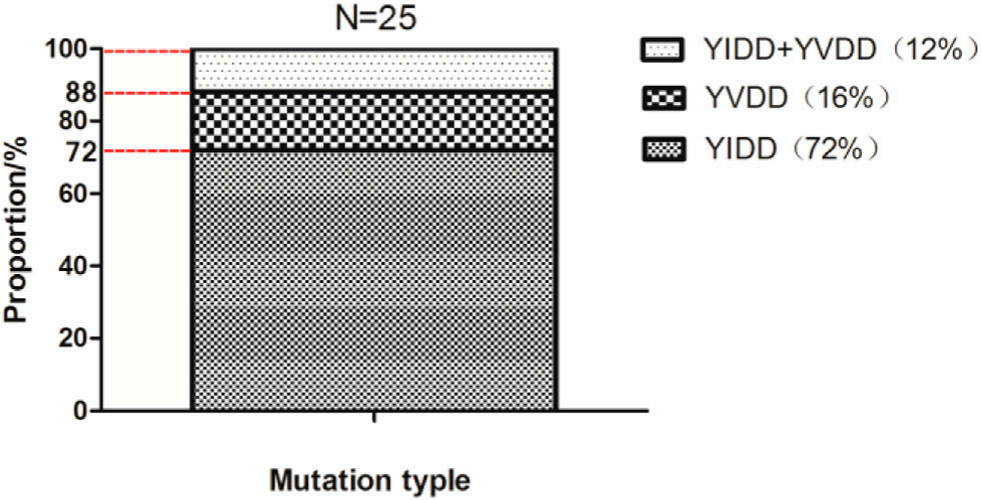
The distribution proportion of YMDD mutations after LAM therapy in CHB patients.

YMDD variants were found in 30.12% (25/83) of CHB patients. Of the YMDD mutations, YIDD, YVDD and YVDD + YIDD were found in 18 (72%), 4 (16%) and 3 (12%) patients, respectively (Table.1; Figure 1). The prevalence of reverse transcriptase rt204I mutants (YIDD variant, 72%) were significantly higher than rt204V mutants (YVDD variant, 16%) and hybrid mutation (YIDD + YVDD variant, 12%) in CHB patients (*p* < 0.05).

### Time for Variation Tendency of YMDD Mutations

**FIGURE 2:**
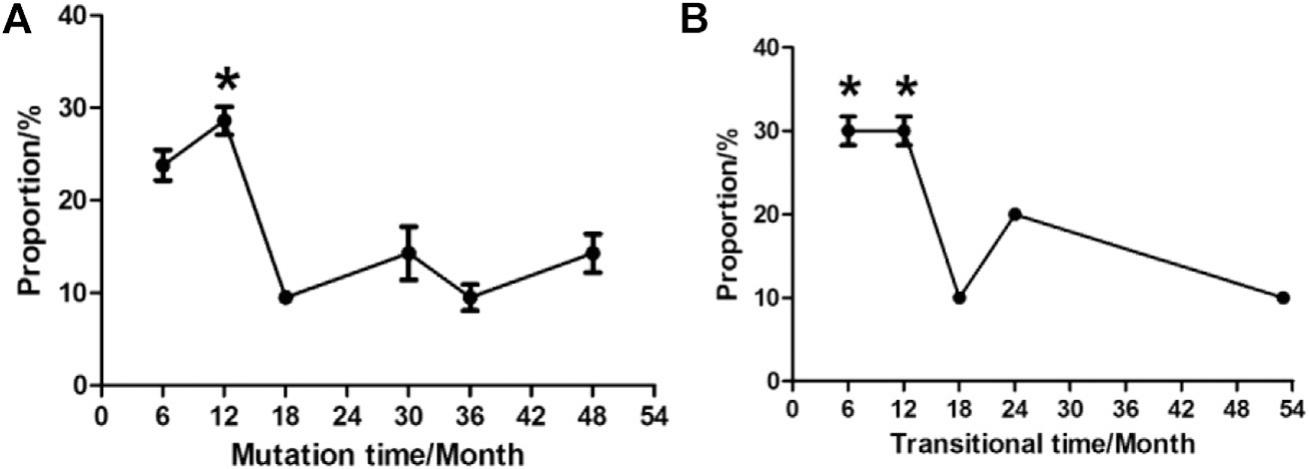
Times of variation tendency of YMDD gene mutation during LAM therapy in CHB patients. **(A)**: Time of mutation change from wild type into mutant type after LAM therapy. **(B)**: Time of transition from mutant type into under detection after therapy.

The time for variation tendency of YMDD gene mutation during LAM therapy in CHB patients was showed in Figure 2. 52.4% CHB patients developed YMDD mutation less than 12 months after LAM therapy (*p* < 0.05) when comparing with other patients. Interestingly, 14.3% patients’ YMDD mutations occurred after4 years post LAM therapy. Besides, our study showed that 60% mutational patients were under the limited detection less than 12 months after therapy of LAM combined with adefovir dipivoxil (ADV) or telbivudine (TBV) (*p* < 0.05, Figure 2B).

### Relationship Between HBV DNA Level and Different YMDD Mutation Status

**FIGURE 3:**
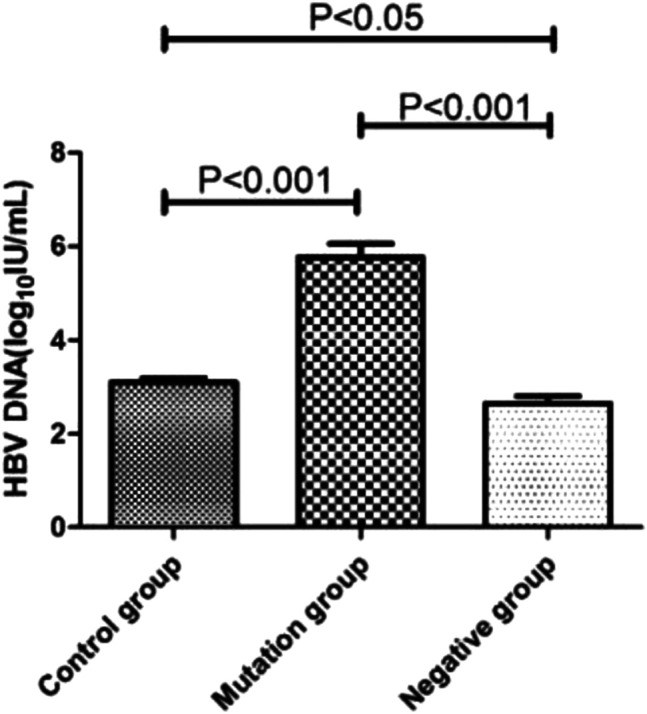
HBV DNA level in different YMDD mutational motif and control groups.

The HBV DNA level of control group, mutation group and negative group were 3.10log_10_ IU/mL, 5.77 log_10_ IU/mL and 2.63 log_10_ IU/mL, respectively (Figure 3). The level of mutation group were significantly higher than control and negative group (*p* < 0.001). What’s more, the control group’s HBV DNA load (3.10log_10_ IU/mL) also higher than the negative group (2.63 log_10_ IU/mL, *p* < 0.05).

### Relationship Between HBsAg, HBeAg, HBeAb Status and YMDD Mutations Patterns

**FIGURE 4:**
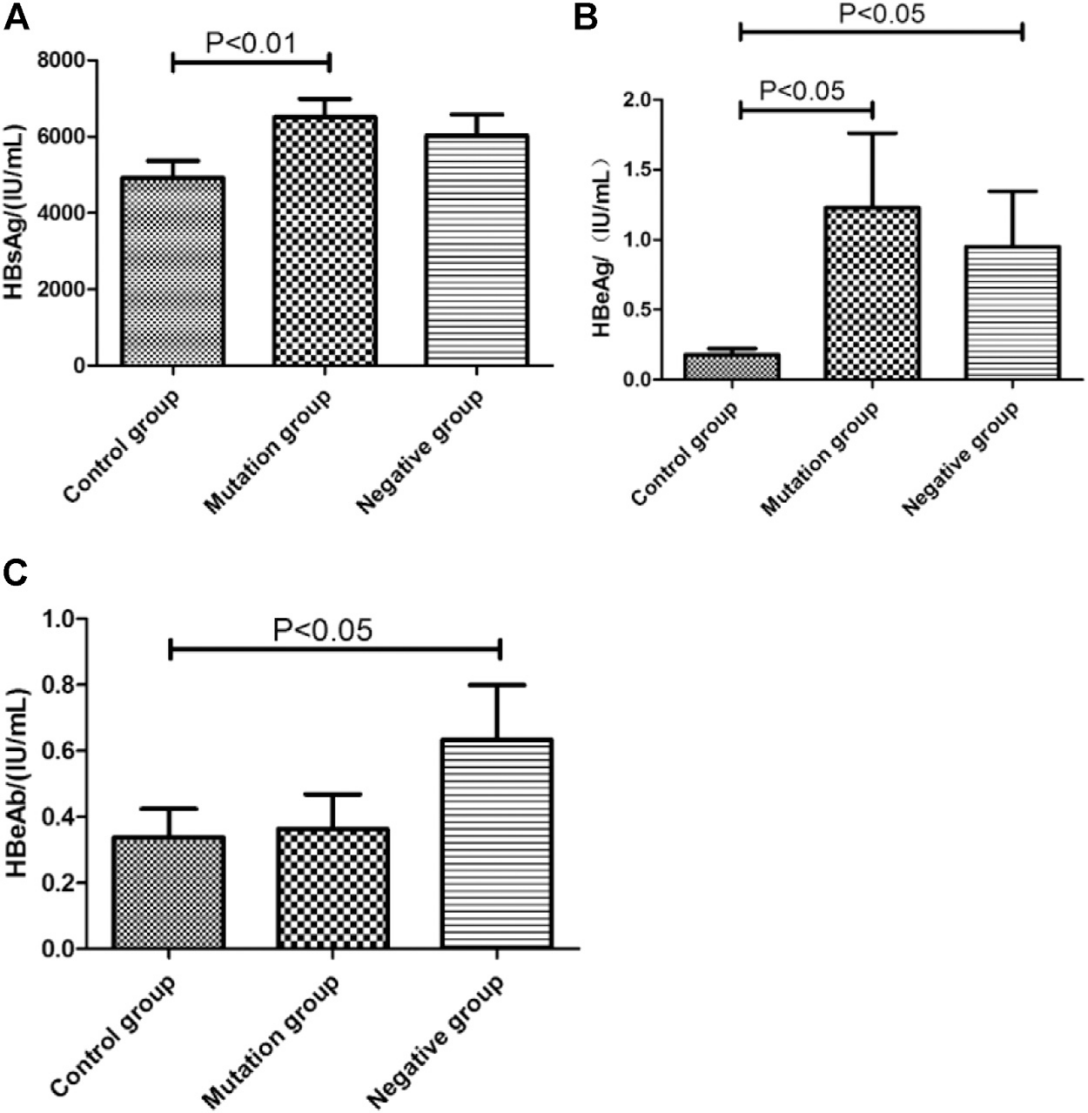
The virologicalof HBV marks level in different YMDD mutational motif and control groups. **(A–C)**: Distribution of HbsAg**(A)**, HbeAg**(B)**, HbeAb**(C)** titers in control group, mutation group and negative group, respectively.

Status of serum HBV marks (HbsAg, HbeAg, HbeAb) in control group, mutation group and negative group were detected (Figures 4A–C). Both load of HBsAg (6,468.75 IU/ml, *p* < 0.01) and HBeAg(1.23 IU/ml, *p* < 0.05) in mutation group were higher than control group (HBsAg:4,898.89 IU/ml, HBeAg:0.1 IU/ml). Comparing with control group, negative group had higher level of HbeAg (*p* < 0.05). Interesting, the load of HbeAbin negative group (0.45 IU/ml) were higher than control group (0.27 IU/mL, *p* < 0.05).

### Association Between ALT, AST Levels and YMDD Mutations Patterns

**FIGURE 5:**
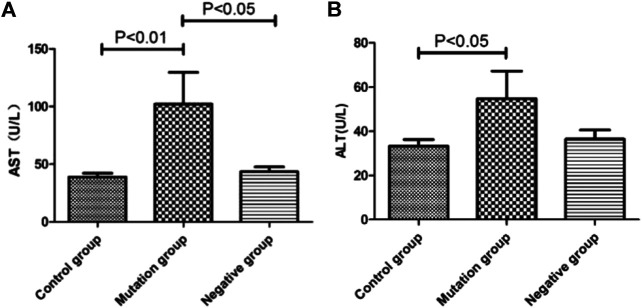
The level of ALT and AST in different YMDD mutational motif and control groups. **A. B:** Biochemical parameters of ALT**(B)** and AST**(A)** associated with/without YMDD mutations in different groups.

Biochemical parameters of ALT (*p* < 0.05) and AST (*p* < 0.01) in mutation group were higher than control group, respectively (Figures 5A,B). What’s more, the level of AST in mutation group (55.45 U/L) were also higher than negative group (41.46 U/L, *p* < 0.05, Figure 5A).

## Discussion

The emergence of YMDD mutations is the main problem after LAM treatment in CHB. YMDD mutations is the C domain of the HBV DNA polymerase gene mutation. No adefovir-associated resistance mutations were identified in the HBV DNA polymerase gene, so ADV (Adefovir Dipivoxil) is an optional therapy for LAM-resistant patients (Marcellin et al., 2003). In spite of lots of studies focusing on the YMDD mutations, the clinical course of hepatitis B in patients with lamivudine-resistant mutants is variable and the long-term outcome remains to be determined.

In this study, our results showed that YMDD mutation patterns of YIDD, YVDD and YVDD + YIDD were found in 18 (72%), 4 (16%) and 3 (12%) patients, respectively (Table.1; Figure 1). Consistent with our results, Kim et al. found that rtM204I, 45 (57.7%); rtM204V, 26 (33.3%); and rtM204I/V, 7 (9.0%) (Kim et al., 2012) Similarly, Pan et al., reported that YIDD mutation is the vital mutant in lamivudine-resistant HBV mutants, which is more than double of the YVDD mutation (Pan et al., 2007). YVDD mutation is more common in genotype A (Zöllner et al., 2004). This result primarily represented the main YMDD mutation patterns after LAM treatment. The underlying mechanism for different prevalence of the mutational patterns in different HBV genotypes should be further investigated. Few studies were investigated in terms of the time during variation tendency of YMDD mutations. Our results showed that 28.6% HBV patientsdeveloped YMDD mutation less than 12 months after LAM therapy (*p* < 0.05, Figure 2). Interestingly, 14.3% patients developed mutations more than 4 years later after LAM therapy (Figure 2). This might be due to that the patients are consisted of different CHB genotypes in the current study. Patients’ viral load is the factor that is admitted by majority to be correlated with mutation rate.

However, further studies are needed to elucidate the real reason. Besides, our study showed that 30% mutational patients were under the limited detection less than 12 months after therapy of LAM combined with Adefovir dipivoxil (ADV) or telbivudine (TBV) (*p* < 0.05, Figure 2B). It will be meaningful to elucidate the dynamic status of YMDD mutants during the LAM treatment. These findings may have impacts on the clinical course of the patients.

YMDD mutants were found to be attenuated in replication capacity and pathogenicity (Tan et al., 2012). The incidence of YMDD mutations may be correlated with the HBeAg status and the HBV DNA level (Tan et al., 2015). It is valuable to clarify the relationship between the occurrence of YMDD mutants and HBV DNA level. Consistent with studies found HBV DNA level might have a positive correlation with YMDD mutations (Tan et al., 2015). Compared to control (3.10log_10_ IU/mL) and negative (2.63 log_10_ IU/mL) group, the HBV DNA level were significantly higher in mutation group (5.77 log_10_ IU/mL, Figure 3). Some studies also found HBV DNA levels were lower than baseline after emergence of YMDD mutants (Pan et al., 2007). But authors investigated the viral differences among genotypes B and C *in vivo*. YMDD mutational patterns may relative to different genotypes. YVDD type tends to have higher levels of HBV DNA than YIDD type *in vitro* and *in vivo* (Tacke et al., 2004). What’s more, the control group’s HBV DNA load (3.10log_10_ IU/mL) were also higher than the negative group (2.63 log_10_ IU/mL, *p* < 0.05). This may due to the mix real normal and in the control group.

HBV DNA levels and HBeAg status are independent factors associated with the emergence of rtM204 I/V Pan et al. (2007). We found status of serum HBV marks HBsAg (6,468.75 IU/ml, *p* < 0.01) and HBeAg(1.23 IU/ml,*p* < 0.05) in mutation group were higher than control group (HBsAg:4,898.89 IU/ml, HBeAg:0.1 IU/mL, Figure 4A,B). HBeAg represent replication capable of HBV. Consistent with that HBV DNA level were significantly higher in mutation group than control group (Figure 3). Comparing with control group, negative group had higher level of HbeAg (*p* < 0.05, Figure 4B). Interesting, the load of HbeAb in negative group were higher than control group (*p* < 0.05, Figure 4C). After LAM treatment, immune system will be more effective to clean virus in non- YMDD mutations than mutations patients. Results also suggested that HBV-DNA levels and HBeAg status can use as reference for chronic HBV infection lamivudine treatment in clinic.

Currently, there is no evidence that YMDD mutations are associated with ALT level (Tan et al., 2012). In this study, we found ALT (*p* < 0.05) and AST (*p* < 0.01) in mutation group were higher than control and negative group, respectively (Figures 5A,B). YMDD mutants may worsen patients liver function. After the emergence of YMDD mutants, lamivudine treatment can improve liver injury. Some studies had found no significant difference in the serum ALT normalization between the patients with each major mutation patterns (Cha et al., 2009). But Kim, et al. had found statistically significant differences in serum ALT between the rtM204I and rtM204V + rtM204I/V mutation groups at 6 and 12 months after the initiation of ADV add-on LAM combination treatment (Kim et al., 2012). Also some studies had found HBV DNA and ALT levels of patients with YMDD mutations at the end of follow-up were lower than that at the baseline (Liaw, 2001). But the underlying mechanism has not been confirmed.

Considering the YMDD mutations could be acquired from other people instead of occur spontaneously. Further studies are needed to distinguish acquired mutation from spontaneous mutation. What’s more, Large-scale population and more samples studies in multi countries are necessary to evaluate the influence of YMDD mutations in hepatitis B progression and antiviral treatment.

## Conclusion

Prevalence of YMDD mutation patterns are different. The time course during the YMDD mutations were different. Serum hepatitis B virus (HBV) DNA levels after YMDD mutants were significantly higher than control and negative group. Serum HbsAg and HbeAg in YMDD mutants were also higher than control and negative group, but have no obviously difference in HbeAb. ALT and AST levels also showed significantly higher in mutants group.

## Data Availability

The original contributions presented in the study are included in the article/Supplementary Material, further inquiries can be directed to the corresponding author.

## Abbreviations

CHB, chronic hepatitis B; YMDD, tyrosine-methionine-aspartate-aspartate; LAM, Lamivudine; ADV, adefovir dipivoxil; TBV, telbivudine; HCC, hepatocellular carcinoma; HBeAg, hepatitis B e antigen; HBV DNA, hepatitis B virus DNA; HBsAg, hepatitis B surface antigen; HBV, hepatitis B virus; HCV, hepatitis C virus; HDV, hepatitis D virus; HIV, human immunodeficiency virus
